# Choroidal thickness changes in post-COVID-19 cases

**DOI:** 10.5935/0004-2749.2022-0165

**Published:** 2025-08-21

**Authors:** Salih Uzun, Fatma Uzun

**Affiliations:** 1 School of Medicine, Stanford University, Stanford, California, USA; 2 Department of Pediatricsd, Ankara Sehir Hastanesi, Ankara, Turkey

Dear Editor,

We have read and reviewed the article entitled “Choroidal thickness changes in post-COVID-19 cases” by Konuk et al. with great interest^(1)^. The authors evalua ted the effects of the coronavirus disease-2019 (COVID-19) on the choroidal thickness (CT) using enhanced depth-imaging optical coherence tomography (OCT).

The authors reported that post-COVID-19 cases exhibited a significantly thicker choroid when compared to healthy subjects at the subfovea, 500-µm temporal to the fovea, and 500- and 1000-µm nasal to the fovea (p=0.011, p=0.043, p=0.009, and p=0.019, respectively). They found that CT was increased in post- COVID-19 patients, possibly in relation with inflammation associated with the pathogenesis of COVID-19.

We express our gratitude to the authors for this valuable study. However, we would like to request Konuk et al. to clarify some important points that may affect CT measurements and the results of the present study.

The choroid is one of the most vascularized regions of the human body. Therefore, various local and systemic physiologic/pathologic conditions and environmental factors affect CT. The literature expounds that age, sex, systemic/local diseases and their treatments, use of medicine, intraocular pressure, refractive error, and several other factors affect CT^(2)^. In addition, body mass index, menstrual cycle, and systemic blood pressure have a remarkable effect on CT. Moreover, CT exhibited shows considerable diurnal variation and a choroid can increase its thickness by 50% in an hour and by 4-times in a few days^(3)^. Furthermore, consuming food and caffeinated and/or non-caffeinated beverages and even exercising before OCT measurements can induce significant changes in CT^(2)^. We would like to ask authors whether all these factors were assessed during data collection and extraction in their study.
