# Supplementary material for: Choroidal thickness changes in post-COVID-19 cases
Source: Arq Bras Oftalmol. 2025 Aug 21;85(4):430. doi: 10.5935/0004-2749.2022-0165 (PMC11878406; doi:10.5935/0004-2749.2022-0165)
Supplement: Related texts

# Reply: Choroidal thickness changes in post-COVID-19 cases

**Authors:** Serífe Gülham Konuk

**Affiliations:** 1 Department of Ophthalmology, Gaziosmanpasa University Medical Faculty, Tokat Gaziosmanpasa University, Tokat, Turkey

Dear Editor,

First, we would like to thank the authors for their interest in our study.

As mentioned, the choroid is one of the most vascularized regions of the human body. Therefore, various local and systemic physiologic/pathologic conditions and environmental factors affect the choroidal thickness^(1)^. We reported in our study that there was no significant difference between the study groups in terms of age and gender (p=0.063 and =0.51, respectively)^(2)^. We evaluated the study files and added the missing portions of the results. No participant showed any systemic/local disease without COVID-19 disease in the study. There was a history of anticoagulant and antiviral drug use in the Covid group. However, to the best of our knowledge, no study has yet evaluated the effect of these agents on the choroidal thickness.

Comparison of the intraocular pressure and spherical equivalent between the groups revealed no statistically significant difference (p=0.15 and =0.21, respectively).

Our measurements were made between 10 and 12 AM to reduce the effects of diurnal variation on the choroidal thickness.

However, the participants were not questioned about their body mass index, menstrual cycle, caffeine use, and food consumption in our study, which can be considered as a limitation of the study. We thank the authors for pointing out the deficiency in the article and their valuable contribution.

## References

[1] Nickla DL, Wallman J. (2010). The multifunctional choroid. Prog Retin Eye Res.

[2] Konuk SG, Kilic R, Turkyilmaz B, Turkoglu E. (2022). Choroidal thickness changes in post-COVID-19
cases. Arq Bras Oftalmol.
